# Localised transmission hotspots of a typhoid fever outbreak in the Democratic Republic of Congo

**DOI:** 10.11604/pamj.2017.28.179.10208

**Published:** 2017-10-27

**Authors:** Engy Ali, Rafael Van Den Bergh, Rob D’hondt, Donat Kuma-Kuma, Anja De Weggheleire, Yves Baudot, Vincent Lambert, Paul Hunter, Rony Zachariah, Peter Maes

**Affiliations:** 1 Médecins Sans Frontières - Operational Centre Brussels, Medical Department, Operational Research Unit (LuxOR), Luxembourg, Brussels, Belgium; 2 Médecins Sans Frontières - Operational Centre Brussels, Kinshasa, DRC; 3 Ministry of Public Health, Health District Kikwit, Kikwit, Bandundu, DRC; 4 Network for Application & Development of Aerospatial Remote sensing (N.A.D.A.R), Belgium; 5 Médecins Sans Frontières - Operational Centre Brussels, Medical Department, Brussels; 6 School of Medicine, Health Policy and Practice, University of East Anglia, Norwich, United Kingdom

**Keywords:** Typhoid fever, military camps, Democratic Republic of Congo

## Abstract

**Introduction:**

In a semi-urban setting in the Democratic Republic of Congo, this study aims to understand the dynamic of a typhoid fever (TF) outbreak and to assess: a) the existence of hot spots for TF transmission and b) the difference between typhoid cases identified within those hot spots and the general population in relation to socio-demographic characteristics, sanitation practice, and sources of drinking water.

**Methods:**

This was a retrospective analysis of TF outbreaks in 2011 in Kikwit, DRC using microbiological analysis of water sources and a structured interview questionnaire.

**Results:**

There were a total of 1430 reported TF cases. The outbreak’s epidemic curve shows earliest and highest peak attack rates (AR) in three military camps located in Kikwit (Ebeya 3.2%; Ngubu 3.0%; and Nsinga 2.2%) compared to an average peak AR of 0.6% in other affected areas. A total 320 cases from the military camps and the high burden health areas were interviewed. Typhoid cases in the military camps shared a latrine with more than one family (P<0.02). All tap water sources in both the military camps and general population were found to be highly contaminated with faecal coliforms.

**Conclusion:**

The role of military camps in Kikwit as early hotspots of TF transmission was likely associated with lower sanitary and hygiene conditions. The proximity of camps to the general population might have been responsible for disseminating TF to the general population. Mapping of cases during an outbreak could be crucial to identify hot spots for transmission and institute corrective measures.

## Introduction

Typhoid fever (TF) is a serious public health concern in developing countries, particularly where there is poor sanitation and hygiene. In sub-Saharan Africa, TF was estimated to cause 725 new cases and seven deaths per 100,000 population in 2010 [1]. TF is caused by the bacterium *Salmonella Typhimurium* (S.Typhi), and is transmitted faeco-orally. Without effective treatment, the case fatality rate is 10-30%, with 5-10% developing serious complications [2]. In countries with poor sanitation, TF can be endemic, with intermittent recurring outbreaks. However, documentation of outbreak investigations in such settings is relatively scarce, with most research devoted to mapping of outbreaks in industrialized settings [3]. Moreover, the dynamics of individual outbreaks in urban or semi-urban resource-poor settings in Africa have to our knowledge not been documented. Nevertheless, the analysis of such dynamics and their association with population characteristics is crucial, as they can guide the allocation of resources for effective control programmes and help to develop targeted public health interventions in order to prevent recurrence and/or manage potential future outbreaks [4]. TF has been endemic in the DRC, with repeated and severe outbreaks frequently occurring country-wide [5]. Outbreaks of TF have been reported in the semi-urban context of the city of Kikwit, Bandundu Province, in the year 2006 and 2011. Thereafter, TF has become endemic, with low but persistent numbers of cases reported between and after the two outbreaks. In order to better understand the dynamics of the 2011 TF outbreak in this setting and to assess whether certain areas within the city may represent initial “high risk” zones from where the disease can spread to the population, we assessed: a) the existence of possible hot spots for TF transmission and b) the difference between cases identified within those hot spots and the general population in relation to various characteristics (socio-demographic, hygiene and sanitation practice, and sources of drinking water).

## Methods

### Study design

A descriptive study of the dynamics of TF outbreak, using microbiological analysis of water sources and a structured interview questionnaire

### Study setting

Kikwit was the study site and is the largest city in the Bandundu province, with an estimated population of 400,000 inhabitants in 2011. It is located in the south-west of DRC and is an important commercial and administrative centre. Kikwit health district is supported by one general referral hospital (HGR) and divided into two health zones, north and south. Each health zone (“*Zone de Santé*”) is divided into health areas (“*Aire de Santé*”), each of which is supported by a health post. There are 19 and 22 health areas respectively in north and south Kikwit. There are three military camps in the city for the accommodation of soldiers with their families; with an estimated population of 2400. Geographically, the camps are located in three health areas: Nugubu camp and Nsinga camp are in close proximity to the general population, and Ebeya camp is relatively isolated. The camps have distinct populations and living conditions being a mobile population between different military camps in the country. They originate from various regions of DRC and speak various languages. Generally, the three camps suffer from difficult living conditions in terms of high population density and poor hygienic and sanitation conditions. Due to these specific conditions, for the purpose of this study the three camps were considered as “*de facto*” health areas and were analysed as independent areas. The environment of Kikwit consists of sandy soils with hilltops and plateaus intersected by the river Kwilu and its tributaries. Large erosion gullies run from the hilltops to the river beds. The sources of drinking water include piped water, which is extracted from artesian wells, chlorinated irregularly and distributed through an outdated pipe network to common tap points in the community. Piped water is relatively expensive and difficult to access in large parts of the city, predominantly the south and east parts. For those who cannot afford piped water, there are natural sources (protected and unprotected springs). No changes to the water infrastructure occurred nor major health promotion campaigns were conducted in the intervening months.

### Study population

**Figure 1:**
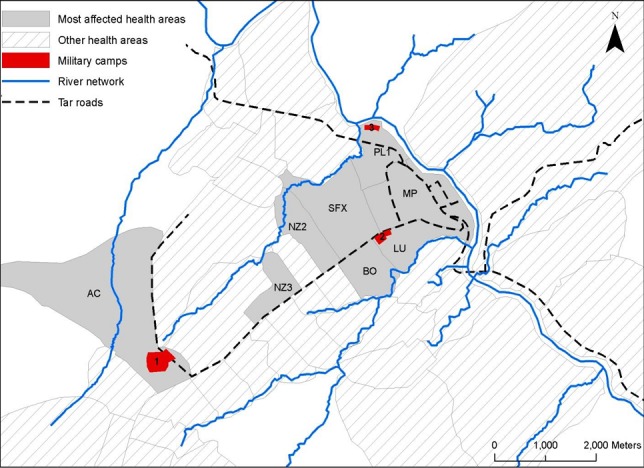
Map of Kikwit city showing the hot spot areas of typhoid fever transmission during the outbreak in 2011, Democratic Republic of Congo. The three military camps are numbered as follows: camp Ebeya (1), camp Nsinga (2) and camp Ngubu (3); the most affected health areas are named as follow: Ancient combatant (AC), Nzinda3 (NZ3), Nzinda2 (NZ2), St François Xavier (SFX), Plateau1 (PL1), Maternite Plateau (MP), Lukolela (LU), and Bongisa (BO)

Included all cases of TF reported during the 2011 TF outbreak. The study was conducted between February and May 2013. The attack rate for each health area was calculated using the denominator from population census data. Eight health areas had an average attack rate (AR) of ≥ 0.6%, while the rest areas had an average AR of ≤ 0.3%. Therefore, an AR of ≥ 0.6% was identified as the threshold for the most-affected health areas and the eight health areas were included; seven were from the north and one from the south (Figure 1). The three military camps were located within three of the included health areas.

### TF outbreak in 2011

The case definition was set by the Ministry of Public Health (MOPH); “*suspected case*” was any person with fever ≥ 38°C for more than three days and digestive disorders (diarrhea, constipation, abdominal pain) and has a negative malaria test. A “*confirmed case*” was a suspected case confirmed by isolation of S. Typhi from blood, bone marrow or duodenal fluid. The outbreak started in the epidemiology week (epi week) 46, the initial laboratory analysis was performed in the HGR laboratory in Kikwit. The results showed that six out of 16 and 7 out of 13, blood and stool culture samples were tested positive for S. Typhi respectively. Then the outbreak was confirmed by the MOPH based on the results of 50 blood and stool samples which were tested in the University Hospital in Kinshasa. Consequently, TF diagnosis was determined based on the clinical definition due to the limited laboratory resources in Kikiwt and the long distance to the laboratory in Kinshasa. Since the beginning of the outbreak, all TF cases had their data entered into a central electronic line list developed by the MOPH which included patient’s name, sex, age, address, date of consultation, signs of TF and, if possible, treatment evolution. The Médecins Sans Frontières (MSF) emergency response to this outbreak included supporting the health district with free-of-charge medical and surgical treatment, training of health personnel and provision of clean water and sanitation.

### Interview survey

In order to understand the potential differences between TF cases identified within the hot spots of transmission and the general population, a semi-structured questionnaire was used to collect information on basic socio-demographic characteristics, knowledge of TF prevention, sources of drinking water, drinking water accessibility, storage capacity and basic sanitation practice. Twelve interviewers and one supervisor who spoke the local languages were trained on questionnaire procedure and the recording of household’s location and its principal water source using hand-held Global Positioning System (GPS) devices. The questionnaire was pre-tested among 20 respondents and subsequently adapted to the local context. Using the recorded addresses in the line list, TF cases were traced and interviewed in the community. Respondents were requested to show their household water storage containers, available soap and latrines. For children less than 13 years old, the interview was conducted with the guardian or a family member who was living in the same household and aware of the condition of the child.

### Water quality testing

Water samples of the principal source of drinking water of all interviewed cases were collected by two trained water and sanitation community workers. Replicate tests were done on water samples onsite for Free Residual Chlorine (FRC) levels using the Hannah Photometer. Due to the difficulty to detect pathogens such as Salmonella sp in surface water, faecal bacteria have been used as an indicator for potential S.Typhi. In this study, concentrations of ThermoTolerant Coliforms (TTC) were used as the primary indicators of faecal contamination [6]. Within three hours after sampling, the 50 ml water samples were analysed for TTC contamination by passing the water through 0.45 micron membrane filters (Millipore Corporation, Bedford, Massachusetts, USA) and subsequently incubating the filters on lauryl sulphate media (18 h incubation at 44°C ± 0.5°C) in a Delagua portable incubator (Robens Institute, Surrey, UK). After incubation, the number of yellow colonies were counted and recorded as the TTC contamination levels (number of colonies/100 ml). If the number of colonies was too numerous to count, a value of > 1000 TTC colonies/100 ml was assigned. These TTC levels corresponded to related health risks as: 0 (extremely low), 1-10 (low), 11-100 (intermediate to high), 101-1000 (very high) and >1000 (extremely high) [7]. At least three samples from each water source during different periods of time were analysed, to cover potential variations in water quality throughout the day (morning and evening) as well as variations related to rainfall and dry events. The most frequently occurring (mode) TTC level was included in this analysis.

### Data and statistical analysis

Due to logistic and human resources constrains, a sample of 25% of all cases were set with a power of 94%. Sample was selected randomly from the line list and then checked not to be residing in the same household. Eventually, 320 TF cases’ addresses were found out and interviewed. Survey data was entered and analysed using EpiData software (EpiData Association, Odense, Denmark). Differences between groups were compared using the Chi-square and Fisher’s exact test for categorical variables as appropriate, and Student’s t-test for continuous variables. The level of significance was set at P = 0.05 and 95% confidence intervals (CI) were used throughout. All GPS readings were encoded and visualized using ArcGIS software (Esri, California, USA).

### Ethics

Ethics approval was received from University of Kinshasa, Ministry of Higher Education, Academic and Scientific Research. Written informed consent was sought and obtained for all respondents. Written consents on behalf of children (< 18 years) enrolled were obtained from caretakers/guardians.

## Results

### Pattern of the TF outbreak and identified hotspots

**Figure 2:**
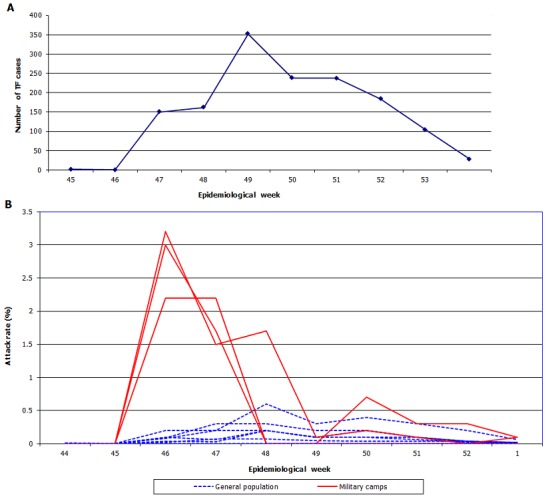
(A) typhoid fever attack rate in all health areas per epidemiological week during the outbreak in 2011, Kikwit, Democratic Republic of Congo; (B) pattern of the typhoid fever outbreak in the military camps and the general population (most affected health areas) in 2011, Kikwit, Democratic Republic of Congo

A total of 1430 TF cases were reported between November 2011 (epi week 44) and January 2012 (epi week 1) (Figure 2A). Of these, 71 severe cases developed peritonitis with perforation. A total of 17 cases died (case fatality rate, 1.5%). The epidemiological curve of the outbreak is shown in Figure 2B. When breaking down the AR per health area, the earliest and highest peak ARs were observed in the three camps during week 46 (Ebeya 3.2%; Ngubu 3.0%; and Nsinga 2.2%), compared to an highest peak AR of 0.6 % in the other affected health areas in week 48 (excluding camps population), suggesting the camps represented the initial focus of TF transmission in Kikwit.

### Typhoid cases in relation to various characteristics of military camps and the general population

**Table 1** shows that out of total 320 TF cases included in the survey, 59 (18%) lived in the camps. The median age of cases in camps and general population is 14 and 13 years respectively. Overall, 75% and 25% of the heads of households in military camps and the general population had an employment contract respectively. The remaining 25% in the camps were either families of deceased army officials or civilians who were living in the immediate surroundings of the camps. University education was significantly higher among the general population than those in camps (15% versus 5%). Almost 75% of cases in the general population and 47% in camps had electricity and/or functioning TV.

**Table 1:** Typhoid fever cases in relation to demographic and socio-economic characteristics in military camps and general population, Kikwit, Democratic Republic of Congo

Variable	Total	CasesMilitary camps	CasesGeneral population	*P* value*
		**n(%)**	**n(%)**	
**TOTAL**	320	59	261	
**Sex**				
Male	165	29 (49)	136 (52)	0.68
Female	155	30 (51)	125 (48)	
**Age** (median, IQR) (year)		13 (8-35)	14 (7-25)	
**Occupation ^a^**				
Contractual worker	109	44 (75)	65 (25)	< 0.001
Owns own business	59	2 (3)	57 (22)	
Occasional daily worker	84	9 (15)	75 (29)	
Other	68	4 (7)	64 (25)	
**Education ^b^ (years)**				
0	22	5 (9)	17 (7)	0.03
1-6	31	9 (15)	22 (8)	
7-11	224	42 (71)	182 (70)	
≥ 12	43	3 (5)	40 (15)	
**Economic status**				
Having electricity and/or TV	224	28 (47)	196 (75)	<0.001
Owning radio	203	34 (58)	169 (65)	0.30
Owning mobile phone	286	50 (85)	236 (90)	0.20
**Topography of house**				
On a lower/bottom zone	23	19 (32)	4 (1)	< 0.001
On a slope	72	2 (3)	70 (27)	
On a crest	225	38 (64)	187 (72)	
**Type of house**				
Brick construction	156	45 (77)	111 (42)	<0.001
Mud construction	164	14 (23)	150 (58)	

IQR: Inter-quartile range

a, b occupation and education of the head of the household

* Chi-squared test for trend.

### Typhoid cases in relation to sanitation and hygiene practices in military camps and general population

**Table 2** shows that all interviewed cases reported the use of latrines for defecation. The practice of sharing a latrine with more than five families was higher among cases in the camps (8% versus 3%). None of the observed latrines of the cases in camps and only 3% of cases in the general population had materials to improve hand hygiene (soap, mud, ash) or water for washing hands at a close distance (< 3 metres) from latrines. Upon request, 66% and 82% of cases in camps and general population showed the available soap in the household. Almost 85% and 82% of cases in the camps and general population knew at least one measure to avoid TF after the outbreak respectively. There was no difference between the two groups as regards to the knowledge of ways to avoid TF and the majority of both groups frequently reported the measure of hand washing.

**Table 2:** Typhoid fever cases in relation to sanitation and hygiene practices in military camps and general population, Kikwit, Democratic Republic of Congo

Variable	Total	CasesMilitary camps	CasesGeneral population	*P* value*
		**n(%)**	**n(%)**	
**TOTAL**	**320**	**59**	**261**	
**No of families sharing latrines**				
1	160	26 (44)	134 (51)	0.02
2-4	149	28 (48)	121 (46)	
≥ 5	11	5 (8)	6 (3)	
**Type of latrines**				
Pit latrine	307	59 (100)	248 (95)	0.08
Flush latrine	13	0	13 (5)	
**Observation of Latrines**				
Water & material for hand washing	7	0	8 (3)	0.51
**Observation of Soap**				
Available soap in household	253	39 (66)	214 (82)	0.006

* Chi-squared test for trend and Fisher exact test used all through for small values

### Typhoid cases in relation to sources of drinking water in military camps and general population

**Table 3** shows that the majority (96%) of cases in the general population used taps at communal distribution points as their principal source of drinking water, while the individual camps each used a different type of source. The principal source of drinking water for cases in camps was an artesian well for Ngubu camp (36%), taps at communal distribution points for Nsinga camp (34%), and an unprotected source for Ebeya camp (30%).Table 4 shows that all tap water sources had an average level of FRC between 0-0.04 mg/L, which is far below the target value of 0.2-0.5 mg/L. All water sources were found to be contaminated with faecal coliforms to a “very high” degree, except for water sources in Plateau 1 and the artesian well in Ngubu camp, which had intermediate to high contamination and no contamination respectively. The majority of cases in the camps (41, 68%) and general population (237, 91%) used the principal source of water for activities such as cooking, washing dishes and bathing. The mean in-house water storage capacity was higher among cases in the general population (170 L) than among those in camps (111 L). Additionally, the practice of covering water containers was much less pronounced among cases in the camps (53% versus 84%).

**Table 3:** Typhoid fever cases in relation to sources of drinking water in military camps and in general population, Kikwit, Democratic Republic of Congo

Variable	Total	CasesMilitary camps	CasesGeneral population	*P* value*
		**n(%)**	**n(%)**	
TOTAL	320	59	261	
**Principal source of drinking water**				
Taps (household)	1	0	1 (0.5)	<0.001^a^
Taps (communal distribution)	271	20 (34)	251 (96)	
Protected spring	4	0	4 (1.5)	
Unprotected spring	21	18 (30)	3 (1)	
Artesian well	23	21 (36)	2 (1)	
**Reasons of choosing principal source^b^**				
Ease of accessibility	259	42 (71)	217 (83)	0.03
Affordable	36	23 (39)	13 (5)	<0.001
Treated/protected	96	7 (12)	89 (34)	<0.001
**Secondary source of drinking water**				
Taps (communal distribution)	100	10 (17)	90 (35)	<0.001^c^
Protected spring	92	3 (5)	89 (34)	
Unprotected spring	66	20 (34)	46 (18)	
Artesian well	22	17 (29)	5 (2)	
Well with/without a hand pump	20	4 (7)	16 (6)	
Surface water	13	3 (5)	10 (4)	
Street vendors	7	2 (3)	5 (2)	
**Observation of water containers**				
Capacity of containers (Mean- Litres)		111	170	<0.001
Principal container covered	249	31 (53)	218 (84)	<0.001
Principal container with narrow neck	315	56 (95)	259 (99)	0.04

a More than one response by the same participant

b Tested taps, springs and artesian wells

c Consider to be approximate as 4 4 of 14 cells have an expected value of <5

* Chi-squared test for trend and Fisher exact test used all through for small values.

**Table 4:** Results of testing water quality at principal sources of drinking water used by typhoid fever cases per health area in Kikwit, Democratic Republic of Congo

Health area	Mean FRC (mg/l)	Mode TTC (no. colonies/100 ml)
Camp Ngubu	0	0
Plateau 1	0.03	11-100
Camp Ebeya	0	101-1000
Nzinda 3	0	101-1000
St Francois	0.01	101-1000
Camp Nsinga	0.02	101-1000
Maternte Plateau	0.02	101-1000
Lukolela	0.02	101-1000
Bongisa	0.02	101-1000
Anciens combatants	0.03	101-1000
Nzinda 2	0.04	101-1000

FRC: free residual chlorine (Target values for water with PH up to 8 is 0.2 to 0.5 mg/l); TTC: ThermoTolerant Coliform (number of colonies/100 ml), accepted TTC level “low risk” is up to 10 colonies/ 100ml.

## Discussion

Our study shows that within the eight health areas most affected by the 2011 TF outbreak in the semi-urban setting of Kiwkit, three military camps had the highest attack rates during the initial phase of the outbreak. The distinct characteristics of camp populations being mobile between camps and the poor sanitary infrastructure in camps may render them vulnerable to outbreaks in a TF endemic setting. As these camps are embedded in the city of Kikwit, it is possible that these camps could have been the focal points of acquiring and disseminating TF to the general population. Our results indicate that the drinking water quality throughout all most affected health areas was uniformly inadequate (high faecal coliform counts and inadequate chlorination), and surprisingly one of the only uncontaminated water sources was found within one of the camps. The outdated water supply system in these areas might have played an important role in disseminating typhoid from early cases in camps to the general population. There were a number of findings that merit discussion. First, although, 75% of the head of household of cases within camps had an employment contract (military officials), the general population had a relatively better socio-economic status, with seven out of ten cases having either a functioning TV and/or electricity - proxy indicators of relative affluence. Moreover, attaining a higher education level was more frequent among the cases in the general population than those in camps.

Second, sharing latrines with more than one family is the usual practice in Kikwit. Interestingly, sharing of latrines predominated among cases in the camps, where six out of ten families had to share a latrine with more than two families. Thus, the ownership of a private latrine might be a protective factor in the early phase of the outbreak. However, all observed latrines of cases, both in- and outside of the camps, lacked water, material for hand washing or hand washing station. The same was observed in a study from rural Kenya where most of the population had soap at home but almost none had a designated hand washing station in the household, which may hinder or prevent hand washing [8]. Cases from the camps were less likely to have soap at the household than those in the general population. These findings are consistent with earlier reports [9, 10]. With the low economic status, especially in the camps, the cost of buying soap becomes a hurdle for the majority of families of an average of seven persons. Locally, homemade soaps of palm oil are available at relatively affordable prices. These products need to be promoted locally by health authorities and NGOs. Third, although the majority of cases in both the camps and the general population had narrow necked water containers, considered to be effective in preventing TF [11], these containers were significantly more covered among cases in the general population. In addition, they had a higher average water storage capacity compared to cases in the camps, which might be also a protective factor against acquiring TF in the initial phase of the outbreak.

These observations - poor economic conditions, suboptimal geographic location, poor hygiene practices and water storage capacity - combined with the overall overcrowding and the higher mobility of camp’s populations (including new arrival and returning soldiers from potential TF-affected areas) may have contributed specifically to the initial flaring of TF cases in the camps and then spread to the general population. Although TF is endemic in DRC, it appears to be re-emerging as an endemic illness in Kikwit after the first outbreak in 2006. In such a context, children may not yet have acquired the immunity to TF, and young children in particular are more exposed to infection at schools. Such findings were observed in a study in Uzbekistan [4]; however, in our study being a student was not assessed independently. Our study had a number of limitations. The main drawback was that it was initiated 13 months after the end of 2011outbreak, and thus the questionnaire did not include questions concerning individual hygiene behaviour and practice during the outbreak to avoid recall bias. Besides, it is difficult to verify the accuracy of the clinical case definition used by MOPH to capture TF cases during the outbreak. Additionally, although no major changes were made to the water distribution systems in Kikwit in the period between the outbreak and our study and measures were taken to conduct the study during a similar season of the outbreak (rainy season from October to May), the water source testing results may not be representative of the water quality during the outbreak. We also assumed that the sanitation practice (soap, latrines) and water storage practice (containers) have not changed since the outbreak. Some names and addresses in the line list were mistakenly reported and it was difficult to find cases who had changed their residency or left Kikwit. There was no available information about the movement of soldiers into or in-between camps during the period preceding the outbreak. Finally, due to the close proximity of the two military camps; Ngubu and Nsinga, to the general population, there might be a proportion of civilian cases who were living in the immediate vicinity of the camps who were recorded as being part of the camp population in the line list.

Possible recommendations to prevent future outbreaks could involve improving the overall sanitation and hygiene situation in the identified hotspots of TF transmission including the military camps by rehabilitating latrines and hygiene promotion. Vaccine-based strategies for TF control are recommended for school-age children in endemic countries - in this context, a targeted vaccination in the camps might be an effective approach in resource-constrained contexts [12, 13]. Moreover, an improved drinking water supply network, optimal chlorination of tap water, regular inspection of the chlorination levels, and rehabilitation of unprotected springs and closing latrines located uphill and in relative proximity to frequented water sources are suggested measures to achieve a decrease in the incidence of TF in settings such as Kikwit.

## Conclusion

The role of military camps as early hotspots of TF transmission was associated with poor sanitary and hygiene conditions. The proximity of camps to the general population might have been responsible for disseminating TF to the general population. Mapping of cases during an outbreak would be a vital manner of identifying hot spots of transmission and prioritizing interventions.

### What is known about this topic

- Epidemiology, microbiology, clinical manifestation, and prevention and management of typhoid fever;
- Risk factors associated with TF transmission in endemic context, this includes poor sanitation and hygiene practice.

### What this study adds

- The dynamics of individual TF outbreaks in urban or semi-urban resource-poor settings in Africa have to our knowledge not been documented;
- The study provides results on the role of military camps which embedded within a semi-urban context in the initial flaring of TF transmission in the camps, and then spread to the general population.

## Competing interests

The authors declare no competing interest
